# Supplementary material for: Decoding kinematic information from beta-band motor rhythms of speech motor cortex: a methodological/analytic approach using concurrent speech movement tracking and magnetoencephalography
Source: Front Hum Neurosci. 2024 Apr 5;18:1305058. doi: 10.3389/fnhum.2024.1305058 (PMC11027130; doi:10.3389/fnhum.2024.1305058)
Supplement: Supplementary file 1 [file Data_Sheet_1.docx]

Supplementary Materials

**
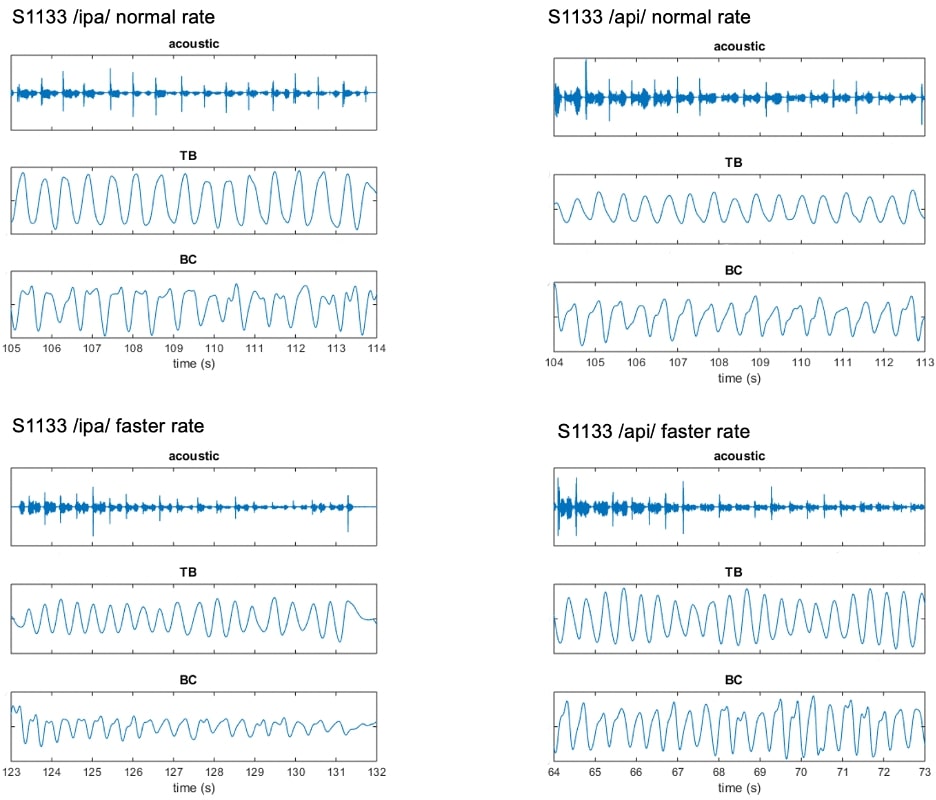
**

Figure S1. Representative acoustic and kinematic measurements from MASK. Data are shown for two participants for a single /ipa/ trial set at normal and faster speaking rates. Shown are (from top to bottom) waveforms for the audio signal, tongue body (TB) gesture, and bilabial constriction (BC) gesture.

**
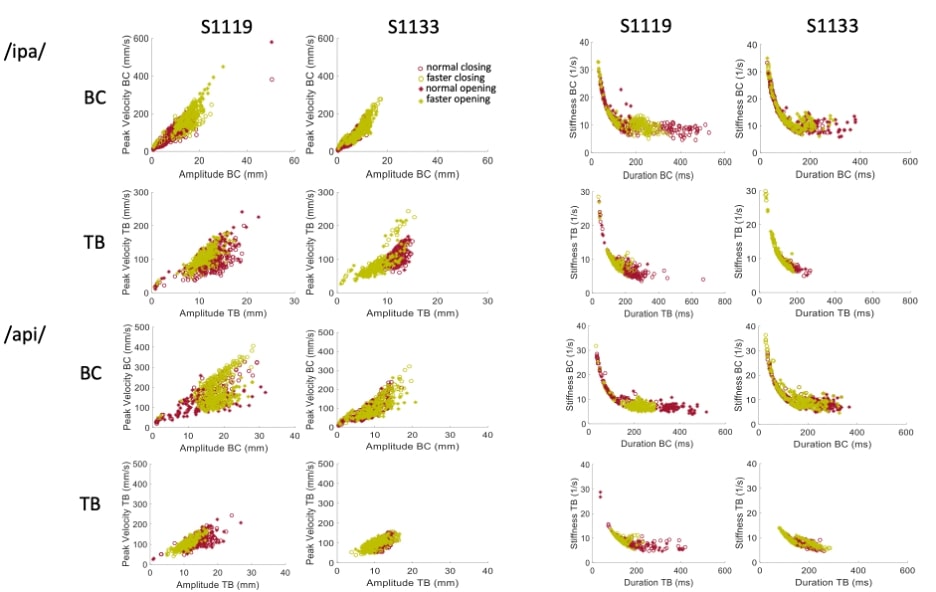
**

**Figure S2. Covariation of kinematic parameters of speech movements for two participants**. Left columns: Velocity versus amplitude. Right columns: Stiffness versus duration. BC = bilabial closure. TB = tongue body.
